# ‘It’s like Going to the Regular Class but without Being there’: A Qualitative Analysis of Older people’s Experiences of Exercise in the Home during Covid-19 Lockdown in England

**DOI:** 10.1007/s41978-020-00078-9

**Published:** 2020-12-02

**Authors:** Lisa Taylor, Jayne Raisborough, Katherine Harrison, Shelly Dulson

**Affiliations:** School of Cultural Studies and Humanities, Leeds Beckett University, Leeds, UK

**Keywords:** Age, Covid-19, Exercise, Home, Zoom

## Abstract

It is expected that the Covid-19 lockdown will have increased physical inactivity with negative impacts for older people, who are at greater risk of health complications from the virus. This paper draws on customer evaluation questionnaire of a Pilates class aimed at people aged over 50 years old, which transitioned from a studio setting to online classes via Zoom at the start of the lockdown in England. The paper aims to (i) evaluate the shift of exercise services to online and (ii) examine how engagement with online services has influenced people’s reaction to Covid-19 and unprecedented confinement to their homes. Our analysis shows that experiences of exercise in the home are dependent on prior exercise engagement, particularly a sense of progress and competency in exercise movements, trust in the instructor and socio-economic privileges that enable participants to love and appreciate their homes. This paper argues that online classes have had positive impact on participants’ ability to cope with lockdown: routine, structure and being seen by others all proved important well-being aspects.

## Introduction

National lockdown, the confinement of people to their homes, became a popular response to Covid-19, with an estimated 4 billion people living in social isolation (Matias et al. 2020). The lockdown in England on 23 March 2020 forced many leisure and sport businesses to suspend activities or to rapidly provide online services. Over lockdown, Zoom, the video-conferencing service, became highly popular as a means of group communication (Sherman 2020). In the UK, Ofcom reported that Zoom usage increased from 659,000 users in January to 13 million by April 2020. It is timely to (i) evaluate the shift of exercise services online and (ii) examine how engagement with online services has influenced people’s reaction to Covid-19 and unprecedented confinement to their homes. We explore these questions in terms of changes to an established business that provides Pilates classes to middle-aged and older residents of a Northern English semi-rural town. Within a week of lockdown, the business, which we call here *Easy Mobilities*, moved from physical classes held in a purpose-built studio, to Zoom Pilates. Asynchronous videos were added to *Easy Mobilities*’ provision a few weeks later. Working with the business owner, we designed a questionnaire to capture the experiences of the clients as they adapted to the changes. As an evaluation, the questionnaire also facilitated clients’ expressions of their satisfaction with *Easy Mobilities*. This research is important to the business because: it can help it understand client behaviour and expectation; reveal any mismatch between perceptions of the client and the business; and help to indicate areas for growth and innovation (Grigoroudis and Siskos 2010).

The research was also motivated by a concern shared by the researchers and business about rates of activity over lockdown. It is widely accepted that regular physical activity is associated with several health and well-being benefits (Herbert et al. 2020). Physical exercise is considered a protective factor for some cancers, stroke, diabetes and cardiovascular disease, and with the delay of up to forty chronic conditions and diseases (Ruegsegger and Booth 2018). Exercise is also associated with reducing anxiety and depressive symptoms and boosting mood, with low-to-moderate regular activity bringing benefits to overall quality of life (Herbert et al. 2020). Exercise is particularly encouraged in mid and later life: exercise that involves balance improvement and muscle strength can significantly reduce the risk of falls, which may cause injury or illness, increase dependency and have long-term effects on social and mental well-being (Aubertin-Leheudre and Rolland 2020). This study focuses on Pilates, a series of low impact exercises that strengthen core muscles to improve posture, mobility, balance and breathing (Joyce and Kotler 2017). Developed by Joseph and Clare Pilates in the 1920s, Pilates been associated with increased cardiorespiratory fitness (Fernández-Rodríguez et al. 2019) and reduction in fall risks and improvements in functional mobility in older people (Roller et al. 2018).

Despite the benefits of exercise, there were pre-Covid-19 global trends of inadequate levels of physical activity for all ages (Guthold et al. 2018), with sedentary lifestyles and lack of activity highlighted as significant public health concerns by the World Health Organization (Aubertin-Leheudre and Rolland 2020). Covid-19 restrictions, such as lockdown, have had an impact: a Sport England report on the activity levels from mid March to mid May found that ‘the number of active adults fell by 7.1%, or just over 3 million, whilst inactivity levels rose by 7.4% or 3.4 million adults’ (Sport England 2020: 3). Dominski and Brandt (2020) argue that lockdown will have increased sedentary behaviour, not least because people will have spent more time consuming a range of online media. This is supported by Ofcom (2020), who reported in June that ‘UK adults are now spending more than a quarter of their waking day online’. While we still need to better understand the links between sedentary lifestyles and screen hours, there is emerging evidence that lockdown has affected health. A recent report has found that one in five people aged 50–70 years declared a decline in physical health since the start of lockdown (Centre of Ageing Better/ Ipsos MORI 2020).

There are several reasons to worry about the decline of health that are specifically related to Covid-19. The first is that exercise can help reduce risks of infections and is reported to help boost immune responses to vaccinations against influenza and pneumonia; inactivity can thus be expected to increase risks of other transmittable diseases in the context of the Covid-19 pandemic (Song et al. 2020). Exercise is also considered vital in dealing with the negative health impacts of social distancing and quarantine. Dominski and Brandt (2020) state that long periods of quarantine are associated with post-traumatic stress disorder (PTSD) and poor mental health: comparisons between people in quarantine for more than and less than 10 days found a marked increase in PTSD symptoms in the former group (Brooks et al. 2020). Older people are more adversely affected in relation to mental health because of the specific ways this pandemic is characterized by ageist discourses that erode self-esteem by seeming ‘to suggest that the death of older people somehow is not as important as loss of life of younger people’ (Brooke and Jackson 2020: 2044). Dominski and Brandt (2020) conclude that inactivity over lockdown has, then, pronounced health risks, particularly for those groups already considered at increased risk from Covid-19. Exercise, particularly taken regularly, can enable ‘a reset of physical and mental well-being’ (Dominski and Brandt 2020). Matias et al. (2020: 871) underscore this point by recommending that exercise should be ‘as vigorously promoted as social distancing itself’.

Given the increased importance of exercise in the context of lockdown, it is important to understand what enables and sustains exercise within the home. We were asked to survey the clients of *Easy Mobilities* Zoom Pilates. The business owner states that *Easy Mobilities* experienced a successful transition to online classes with most studio clients moving online within 2 weeks. This is also the experience of one of the authors of this paper who is a regular client and has found that her usual class cohort has been reproduced online.

Our research had three objectives.

1. To explore what aided the successful transition of studio to streamed online classes;
2. To examine the influence of streamed classes on participants’ experiences of and reactions to lockdown during Covid-19;
3. To explore the affect of weekly or daily streamed classes upon participants’ experiences of home spaces under lockdown and draw conclusions in relation to contextual socio-economic factors.

## Method

Our decision to use an electronic survey was informed by Covid-19. While digital and online methods are now essential ways of engaging with people in lockdown/social distancing, we were also aware that the pandemic has wider impacts: our participants may be experiencing anxiety, loneliness, disrupted family relationships, may have to compete for online access and may have little privacy (Lupton 2020). A survey allowed participants more privacy, more flexibility to respond in their own time and would enable participants to answer how they wished. We also ensured that the survey focused mainly on positive reflections and benefits of their Pilates classes because we realized that normal support systems may not be in place. Our research design and questions were approved by our University ethics processes.

The survey was designed through Survey Monkey, cloud-based software that offers customizable surveys and produces a data analysis report with filter and comparison options (Halim et al. 2018). Survey Monkey surveys are ideally distributed to a targeted population via email and social networks distribution (McPeake et al. 2014). Accordingly, our survey was distributed by *Easy Mobilities* through their weekly e-newsletter on the 16 June 2020 in a segment which explained the research and contained the link (the e-newsletter was already a regular feature, not a special response to Covid-19). This form of distribution was also agreed on because it protected the confidentiality and market-sensitivity of the *Easy Mobilities* client list; we did not have access to the list of clients, nor their level of Pilates engagement, nor did we gather any identifying or personal information. However, it also created a limitation on the data and the scope of the research: we do not know who gave up *Easy Mobilities* when it moved online or why, because they were likely to not have been receiving the newsletter sent to existing clients or if so, they may be among the number who did not respond. The survey ran for two weeks and closed on 30 June 2020. The newsletter was sent to 95 clients, 48 of whom responded to the invitation, giving us a response rate of 51%.

The survey gathered socio-demographic information concerning the respondents’ gender, sexuality, ethnicity and disability. We added questions around age, disability and lockdown status because these categories are pertinent in the context of Covid-19. To understand the transition from studio classes to Zoom Pilates, we asked if participants were *Easy Mobilities* clients before lockdown. We also asked participants to share their thoughts on pre-lockdown, studio Pilates. As such, our survey drew on ‘before and after’ research design used to evaluate the impact of change or intervention (Mills et al. 2010). Traditionally, before and after research gathers data at points pre and post the change. However, as this research was a response to the conditions of Covid-19, we could only ask participants to reflect on their pre-lockdown experiences. We must be aware then, that our participants were engaging with our survey whilst under lockdown and this may have affected their responses to the survey. Apart from the socio-demographic questions, the survey used opened-ended questions.

The qualitative responses were analysed by drawing upon Braun and Clarke’s (2006) step framework for thematic analysis. Braun and Clarke stress data familiarity as the first approach to thematic analysis; accordingly, once the qualitative responds were complied, they were read through a number of times, before the last author producing an initial list of codes, which was shared and discussed across the team to reach a consensus. We used the codes to generate themes from the data, which were reviewed and redefined in turn.

## Participants

Our participants were long term clients of *Easy Mobilities,* with the majority, 83%, being members for over twelve months, the limit of our questionnaire range, with other open data suggesting a membership that ran over years. Only 15% had been members for between seven and twelve months. No one had been a member for less than 3 months and all had studio classes before lockdown. All were able then to reflect on how Zoom Pilates may compare to studio Pilates.

The survey was overwhelming made up of people identifying as women (85%) and 13% as male (2% did not complete the question). Without access to client lists, we can only suspect that this is a good reflection of the gender balance in *Easy Mobilities*, because of reported gender differences in exercises and class participation elsewhere (van Uffelen et al. 2017). When asked to describe ethnicity, 92% of respondents were White British from UK countries. The response population was not ethnically diverse with 2% being mixed race and 2% from non-UK white backgrounds (4% declined to answer). However, these figures are representative of the locality in which the survey was conducted where white ethnic groups account for 94% of the local population, and mixed-race ethnicity accounts for 1% per head of population according to 2011 census data. Participants were able to provide their age as an open response question. The mean (average) age of respondents was 63. The majority of respondents were aged between 57 and 67 years which accounted for 40% of those surveyed. Only two respondents considered themselves to have a disability (4%).

The majority (83%) of respondents reported that they were in lockdown with other people. Of that number, 60% were in lockdown with a one other person (often a partner or spouse) and 21% with children and a partner. One person reported being with wider family members. Those alone in lockdown accounted for 15% of those surveyed (2% declined the question).

## Findings

To understand the transition from studio classes to Zoom Pilates, we had to first appreciate what it was that people valued about the Pilates experiences before lockdown. Thematic analysis generated the following from questions directed at participants’ experiences of studio Pilates.

### Studio Pilates

The biggest benefit to the studio classes was the interaction with the instructor. Participants tied the expertise of the instructor to the instructor’s ability to observe the class, to know their bodies and to correct their performance of the exercises: ‘Exercise tailored to my personal needs ... and ensuring that I did things correctly.’ Being corrected/doing exercise correctly was repeatedly associated with ‘being with’ the instructor in the studio, as is demonstrated by this quotation: ‘Being with an instructor who could make sure I was completing the movement correctly’.

Participants made specific mention of the benefits of Pilates itself in the studio. They spoke of suppleness, flexibility, balance, fitness and core strength, often speaking of their own progress and competency in performing the exercises. Participants also liked aspects of physical space, citing the ability to move about (although there were two comments that studio classes could sometimes be crowded), the use of wall-to-wall mirrors to check their postures and the availability of equipment (mats, bands, balls and blocks). These points were also characterized by ‘doing things correctly’. Feelings about the space in the studio were important too. Others liked that it was a ‘different space’, which suggested a positive contrast with work and home spaces. The physical class was routinely scheduled in a 6-week timetable; participants stressed that this enabled time to access the studio and helped them set aside time to ‘focus on physical activity’.

However, it was the *social aspect* of attending Pilates classes in the studio that was highlighted by participants. They enjoyed the ‘light-hearted culture’ of the classes, mentioning ‘camaraderie’ with ‘like-minded people’. For some, the studio classes ended in an end-of-class get together: ‘the camaraderie, joking, laughing, social contact and coffee afterwards with other members of [the] Pilates class’.

This suggests that both social (friendly) and physical aspects (exercise competency) of studio classes were valued across the sample**.**

## Zoom Pilates

*Easy Mobilities* ended its studio classes following Government advice in March and offered a range of classes over Zoom. To ease the transition, clients continued to pay their normal rate for a single weekly class but could now access daily Zoom Pilates sessions. It was significant that most clients had computer skills: *Easy Mobilities* communicated with clients through email and online booking before lockdown. Four participants reported that they had initial reservations about using Zoom and about the reliability of their internet, but on the whole, the survey indicated a general perseverance with any freezing or sound qualities, with participants seeming happy to have the opportunity to increase their computer competence by using Zoom.

Our survey suggested that participants liked the social aspects and the hands-on instruction that they get from studio Pilates. We might expect that Zoom Pilates would not satisfy these. Our survey asked what benefits our participants derived from Zoom Pilates; we found that people were thankful that the classes continued online, they liked the routine of the classes but also enjoyed the greater flexibility that the online classes enabled.

*Continuity* was the most valued aspect of Zoom Pilates. Over 60% of respondents stated or alluded to it through terms such as ‘keeping up’ and ‘maintaining’ their exercise. ‘Being able to keep up progress that I had made and to remain flexible’ and ‘being able to maintain all the hard work I’d put in over the last 6 years’ are indicative of the quotes that show the importance participants placed on progress. We suggest that the risk of losing physical competency and flexibility was key to participants moving online. Several participants underlined this by reporting that they would have likely been demotivated over lockdown and would have abandoned Pilates: ‘I like the fact it is still going to be honest; I am not sure that I would have kept it up and gone back’.

Yet, the social aspects were also considered valuable. The theme of continuity also speaks to the participants’ enjoyment of maintaining a relationship with the instructor and with other members of the class. Seeing ‘familiar faces’ via the gallery function of Zoom, which shows all the participants, was an important part of maintaining friendships: ‘seeing others’ and also ‘being seen’ helped to create a feeling of still ‘exercising together’. Many agreed with the participant who wrote that she was delighted that she was ‘able to continue with Pilates in my own home but knowing that others are joining in at the same time’.

### Flexibility in Class Availability and Convenience

Zoom classes ran several times a week with clients joining any and as many as they liked over lockdown. Our participants enjoyed this flexibility and indicated that it was an important aspect of their transition to Zoom Pilates. Comments in this theme drew comparisons with studio spaces, with specific mention of difficulties in travelling to the studio and with car parking: ‘not having to travel’ and not ‘finding parking spaces’ were reported as saving, not ‘wasting time’. Overall, however, participants remarked on ‘flexibility in choosing your classes, value for fees and multiple classes per week,’ which allowed people to engage with Pilates even if they had to share computers or physical space and especially for those who continued to work over lockdown.

### The Instructor

Our survey asked participants what they enjoyed about their online classes. Exactly half of those surveyed related their enjoyment and worth directly to the instructor and the nature of the instruction, with many referring to the instructor by name. Again, continuity was emphasized: not only was the instructor’s drive and focus ‘the same as in the studio’ but participants perceived a continuation of teaching quality (‘taught in same way as studio classes with the same focus’). As with studio classes, being seen or watched and corrected were vital aspects to participants’ enjoyment of Zoom Pilates: participants wrote that the instructor ‘is watching you to see you move’ and appreciated the instructor moving into the screen to ‘see you better’. Others repeated the importance of the instructor’s expertise and prior knowledge of her clients: ‘The fact that [the instructor] knows my body and can see me on screen to then provide guidance is needed’.

## Getting through Lockdown

We asked if Zoom Pilates had affected how participants coped with lockdown. Only two participants did not think Zoom Pilates helped, from the rest we generated three major themes from the data:

### Routine

Routine was a large part of what helped people to cope. Participants wrote about structure ‘it added structure to the week’ and routine ‘a bit of routine to start the week’. Although participants had a choice of what classes to attend, each class was timetabled in advance. This created a fixed point around which time and other commitments could be managed. These were important because they helped establish connections to what participants described as normal life: ‘it has helped in terms of not feeling as cut off from my usual routine.’ For some, maintaining a routine was a central coping strategy. This was regardless of whether our participants had ‘childcare and home schooling’ or living alone; for the former, Zoom Pilates made time for ‘self-care’ and, for others without family or work commitments, the scheduled class meant something to ‘look forward to’. For those alone, the scheduled class gave ‘purpose’ to a day ‘and ‘helped keep occupied’. Having a structure and keeping to it also enabled a pleasurable exercise of commitment and ‘discipline’; this was demonstrated in responses similar to ‘I feel pleased with myself for making the effort’ and ‘I like the discipline of it and feel better both for having committed to and carried out the exercise.’ There are strong suggestions that Zoom Pilates’ routine influenced and helped order other aspects of lockdown life: in one response, a participant said they would ‘not have been motivated to do much else were it not for Zoom Pilates’.

### Connection to Normal Life

Our analysis suggests that Zoom Pilates allowed participants to maintain connections to outside life and to their classmates. This is consistent with participants valuing the social aspects of both studio Pilates and Zoom Pilates. In specific relation to lockdown, this social aspect increased in importance. There was a recurring value placed on being seen in class as illustrated by this quote:

‘I think it has stopped me going around the bend, someone saying hello, waving, and the headspace, sometimes [the instructor] says your name so you feel you are looked at…’

Zoom Pilates was regarded as one way of keeping ‘in touch’ with others. ‘It has definitely helped by keeping me in touch’. For two participants in lockdown alone, who stated that otherwise they may not have spoken to someone all day, Zoom Pilates was a vital connection to others ‘Not seeing people in person has been difficult but walking the dog and meeting up with everyone at Pilates has helped’. For others, Zoom Pilates formed a part of a portfolio of other connections; having technology and skill was recognized as a ‘blessing’ in lockdown.

‘Technology has enabled us to keep in touch with family abroad, as well as friends and groups locally, and Zoom Pilates has been part of this. I feel a lot of people who do not have access or do not use modern technology -smartphones/internet banking/ card payments/online shopping may have encountered problems during lockdown’.

### Wellbeing

Almost all respondents (96%), said they had positive feelings as a result of their Zoom Pilates class. 19% of these responses contained references to mobility and flexibility, fitness and a general sense of doing the body ‘some good’. Mental wellbeing was more frequently mentioned with 27% of responses referring to it. Participants frequently associated Zoom Pilates with ‘stress relief’ a ‘calming effect’: ‘I always feel calmer with a better sense of perspective on the day’s events after a class’ ‘and a mood enhancer ‘It gives me a boost - especially in the early part of lockdown when I was feeling quite low’. As with previous discussions, participants also stressed the social impact on their wellbeing as illustrated in this quote ‘Really cheered me up - all my Zoom meetings or get-togethers lift my spirits - actually seeing other people to talk to instead of just chatting on phone.’

## Feelings about the Home

Lockdown has confined people to their homes to different degrees. The data suggests that some participants were Key Workers (health care practitioners and teachers in this sample) with some working away from the home, but the majority of our participants were retired or working from home. We were interested in how feelings of the home may or may not have changed over lockdown. Bradbury-Jones and Isham (2020) argue that public safety messaging over Covid-19 idealizes the home as a place of safety. In what they describe as the pandemic paradox’(p. 2047), they remind us that the home can be site of abuse and violence to which women and children are the most vulnerable. Realizing that the home is then, a paradoxical space, we were interested in our participants’ experiences. 38% of participants said their feelings about their home had not changed, 35% said that it had. Across the responses was a reiteration of idealized versions of home; namely comfort, safety, and appreciation of the home itself. Valuing gardens and views of the rural locality were also recurring responses.

### Luck

Participants who experienced no change to their feelings about their home, stated that they had prior enjoyment and appreciation of their home: ‘always enjoyed our home so no change’. Interestingly, participants expressed their enjoyment as the outcome of good fortune or luck (‘still like it [the home] just as much - I’m very lucky’). Luck related to prior housing choice decisions to live in rural places and to have a garden ‘I love my home and haven’t felt trapped at all. I’m fortunate that we live in a rural place so have lots of great views and a garden.’. Our analysis suggests that loving one’s home was also related to the ability to see a rural landscape or get outside.

### Appreciation

Although appreciation ran across both sets of responses, the participants who expressed a change in feeling towards their home indicated a *growing* appreciation of it over lockdown. For those with usually busy lives, lockdown was threatening, yet a different relationship with indoors developed:

‘I'm usually out and about all the time, so this has been very different for me. I actually think I feel a greater ownership of my home, and I enjoy that..”

For Key Workers there was a sense that the home had become a sanctuary: one participant wrote: ‘I have continued to work at the hospital during lockdown and have valued my home environment even more when I come home.’

For others, the garden become increasingly valued. Many repeated these sentiments: ‘I appreciate the garden more than I have ever done’. There was a suggestion that the ability to be in the garden gained importance as lockdown restrictions tightened. One participant stated: ‘as I am shielding and not able to go out even for exercise until very recently, I have appreciated my garden more than ever’. Appreciating a view anew was also a strong feature: ‘I have really appreciated being able to look out at a ‘green’ view - and have a garden.’

Those who developed a stronger appreciation of their homes and gardens, also spoke of using lockdown ‘to sort some things’. Participants spoke of lockdown as an ‘opportunity to get lots of jobs done’, including ‘rearranged wall hangings in some rooms.’ and ‘to make improvements such as tidying up, decorating etc’.

Some responses were detailed and illustrated a stronger shift in feelings:

‘It changed, I felt locked in at the start, I was furloughed and on my own. I had nothing to do and the walls came in bit, the place looked shabby and bit grotty. I did a spring clean, I wanted to decorate but had to wait til I could. I moved things around, order new bedding, not cheap and had to be careful because of furlough… to be honest, I could keep this up.’

There was wider evidence of using the home in different ways or living differently that extended from ‘moving things around’:

I tried to use the rooms to give structure - so no teas on my knee - at the table in the kitchen, then tv room, then bed, to change things up. I really like home now and thought of all the people stuck in awful places, imagine having kids and no space.’

This quote indicates a sometimes tacit comparison with others who lived in smaller, busier homes, often imagined as inner-city high-rise flats without gardens ‘awful places’. This imaginary of what might have been, boosted an appreciation of the home in terms of safety and comfort.

There was also the suggestion that lockdown had caused a ‘calming’ that was key to new appreciation of the home and wider locality. Online exercise was cited as part of this refocusing in the home and helping repurpose it as a place of relaxation and activity:

‘Like many people, lockdown has really made me slow down and appreciate the simple things in life. I've always loved nature, but lockdown made me value and appreciate it even more. I feel very lucky to live in a lovely town and gave a lovely home and garden and enough money. I've enjoyed being able to do as many Pilates classes as I want. I also do a weekly zoom yoga class. I've read more and done more gardening, even growing my own veg!’

‘I'm enjoying being at home more, relaxing and not rushing. I do zoom choir as well as zoom Pilates, both of which work really well from home so I'm happy that I can continue with my two hobbies.’

## Discussion

Our research had three objectives

1. To explore what aided the successful transition of studio to streamed online classes;
2. To examine the influence of streamed classes on participants’ experiences of and reactions to lockdown during Covid-19;
3. To explore the affect of weekly or daily streamed classes upon participants’ experiences of home spaces under lockdown and draw conclusions in relation to contextual socio-economic factors.

In terms of our first objective, our analysis suggests that a successful transition from studio to Zoom Pilates for our sample rested on two main aspects. The first was the existing relationship with the Pilates instructor, which itself drew on the instructor’s Pilates expertise and upon a past trust that the instructor could personalize exercises for her clients’ bodies. Estabrooks et al. (2004: 233) have previously noted how the instructor is ‘the most important determinant of participation in physical exercise groups’. Studies of physical presence exercise classes have demonstrated the importance of the instructor in creating cohort experiences and a sense of group identity (Stevens et al. 2020) and creating confidence and empowering messaging, both of which are important for continued engagement (Chemers et al. 2000). Our study suggests that importance of the instructor carries through into Zoom space although it may demand more explicit performance than in the studio. Our participants felt ‘seen’, particularly when the instructor repeatedly declared herself to be watching and by actively peering into the screen. The instructor’s commentary around the exercises helped people correct their postures. That the instructor called individual class members by their name was a vital part of our participants’ sense that they were ‘seen’.

The second factor was the investment our participants had made in their own progress. Hawley-Hague et al. (2016) argues that older people are more likely to continue an exercise class if they enjoy it and if they perceive improvement. For our sample, exercise competency was hard-earned, in some cases over many years, with clear self-reported benefits. There was a marked reluctance to give this up over lockdown, which helped people learn new technologies (Zoom) and overcome obstacles. Underpinning this was a sense that participants might have given up, felt de-motivated and then, couldn’t imagine themselves returning to post-Covid-19 class. This suggests that losing progress is on the one hand a motivation to move online for class-continuation but may, on the other, prove an obstacle to people’s return to exercise post-Covid19, if no online class was available.

Our second objective was to examine how Zoom Pilates may have helped our participants cope with lockdown. There were two aspects: routine/structure and sociality. It has been suggested that lockdown will have negatively affected daily habits and routines, with consequences for people’s well-being, sleep and mental health (Aymerich-Franch 2020). Our analysis suggests that timetabled classes helped structure time and to give purpose and definition to a day. For those in busy households, a class allowed ‘me-time’ and access to ‘headspace’ and relaxation. We found too that Zoom Pilates enabled a continuity of the social aspect of the classes. Zoom enabled this by allowing all class members to see each other in gallery view – waving and chatting briefly before and after the class. There was also a strong appreciation of doing exercise at the same time with others. Overwhelmingly, however, it was the wellbeing aspects of Zoom Pilates that helped people cope by providing a focus, connection to others and a sense of accomplishment. Added to this were embodied gains from the exercise itself, when participants wrote about feeling stretched or energized, with impacts on their mood.

Our final objective was to explore participants’ perceptions of their homes over lockdown. Lades et al. (2020) researched the emotional effects of location during Covid-19 lockdown. Using a scale between 1 and 7, with 1 referring to little emotional effect to 7 very much, they found that ‘outdoors/nature’ produced the most positive response on the mean affects scale at 5.51, with one’s ‘own home’ coming third after ‘other peoples’ homes’ at 4.14. Yet as Bryne argues: ‘we cannot practice social distancing without a home, and so our new-found interest in social distancing tells us something about the very nature of home’ (2020: 2). In this study, home takes on seismic significance. Respondents offered, at times, extended testimonials about the importance and centrality of home during lockdown. They reflected on how ‘fortunate’, ‘lucky’ and ‘blessed’ they were to be homeowners during the pandemic. In their study on the importance of homeownership in New Zealand, Dupuis and Thorns (1997) draw on Giddens (1984) to argue for the centrality of home to one’s ‘ontological security’. Bryne’s (2020) discussion of staying home in the context of the pandemic builds on this – arguing that home becomes increasingly significant when the wider pandemic beyond the front door holds an unheimlich - or unhomely - atmosphere. Our data revealed that feeling ‘safe’ at home was a central theme, followed by ‘comfort’ and ‘relaxation.’ During the uncontrollable emergency of Covid-19 lockdown, the ontological security of home becomes paramount: it provides a retreat from societal surveillance; it taps in to the rhythms, routines and habits of ‘familiar time/space pathways’ in the corridors and rooms of home and it provides the permanence and continuity that enable the home to become a site where practices of leisure and aestheticization enable people to engage in identity construction. Many of the homes of our respondents were loved - the home is ‘sanctuary’, a space of surfaces and textures far more noticed, worked over and enjoyed. It is also a site inexorably connected to the garden and to views of the surrounding landscape. There is a tradition of writing about the importance of aestheticising the home to make it homely (Blunt and Dowling 2006; van Lanen 2020). We found that several respondents told us that their homes felt more uniquely related to who they were: ‘my home is more ‘me’,’ one respondent told us. In other words, it had become a space where self-identity could be sharpened. Given the importance of the mundane, circadian routines of home for feelings of safety, it should not surprise us that the exercise benefits of regularity, routine and structure of Zoom Pilates could be easily calibrated into the lives of our over 50s, largely female, relatively affluent group. It managed to marry ‘exercise’ (given the highest mean average score in Lades et al. (2020) on positive emotional affects of 5.53) with the comfort of home. There is a lack of research exploring the influences on indoor environments, specifically the home, on older peoples’ exercise activity (Ashe 2018), however, there is some suggestion that the stress-relief of exercise – its restorative capacity – which is normally associated with a new or different place, can also be achieved ‘by perceiving an old environment in a new way’ (Roe and Roe 2018: 486). According to them, more research is needed on ‘the need for visual delight and aesthetic stimulation’ (Roe and Roe 2018: 491) to promote sustainable indoor exercise. We hope our research goes some way to addressing the gap.

However, it is vital that these benefits are understood in the context of intersections of the age, social class, gender and ethnicity of our sample in terms of how these pertain to the experience of home. Consideration of the demographic characteristics of respondents and qualitative data generated from the open questions related to personal experiences and homes revealed that the positive experience of Zoom Pilates was contingent on having access to private, spacious accommodation within which to perform Pilates movements, as well as feelings of pride about the domestic home. We identified a clear middle-class perspective contingent on ‘lovely’ domestic spaces, gardens, picturesque views, peace and quiet. As Bryne argues, for those who rent in precarious circumstances, homeliness cannot be taken for granted; many tenants face a myriad of controls and prohibitions – to paint a wall, to own a pet - the privileges which make a house a home and a home a retreat are beyond reach (2020: 4). It was significant that our participants considered themselves ‘lucky’ when they imagined other ‘awful’ spaces in which they might have lived and when they expressed sympathy for those people in lockdown with little space or views. We are reminded of Sayer’s analysis of middle-class distinction work. Sayer rejected the view that middle class distinction is always and already antagonistic and driven by an urgent need to distinguish from the working classes. For Sayer, class is an embarrassing subject for those with class privilege. Sayer argued that the middle classes wove an awareness of class inequalities as unfair with a defensiveness of their own privileges:

‘There is a widespread sense in lay thought - sometimes articulated, but often only subconsciously felt - that class differences are at least in part unjust, insofar as individuals' position and life chances are a matter of luck, according to the accident of birth. Insofar as actors recognize this injustice - and it is hard for them not to - it prompts mixtures of guilt, resentment and defensiveness, and the balance of these feelings and the ways of handling them are likely to vary according to class position’ (Sayer 2002: 4.3)

We contend that ‘luck’ is one of the ways our participants ‘handled’ these feelings: it allowed compassion for an imagined other, a sense of appreciation for the results of luck, yet still allowed space for social and material privileges to be unacknowledged as consequences of social determinants. Our data prevents us from exploring these further, but there is the suggestion here that examination of the success of online classes over lockdown can offer useful insights into the nuanced and shifting ways class distinction work, in relation to domestic spaces, is done.

What of the future? This is an important question given the uncertainties of both the virus and the current Government’s responses to it. Our survey found that most respondents felt that they had benefited and intended to continue with online exercise classes in the home after lockdown or take a blended approach, combining studio classes with online participation. This brings increased weekly exercise possibilities to users which undoubtedly enhances bodily fitness and, as a result, feelings of wellbeing. Being able to blend the ‘ambience’ of the studio space with the opportunity to choose online classes enabled clients to work beyond a set, shared menu to a more individual ‘MePilates’ based on a nexus of factors they could weigh up and prioritize. In this way *Easy Mobilities* can enable its clients to fashion their own individualized health menu across particular space and time dimensions, while the continued close alignment to home space as the pandemic continues ushers in more effective management of the home and the leisure possibilities located in and around it.
